# Correction: Tissue acidosis induces neuronal necroptosis via ASIC1a channel independent of its ionic conduction

**DOI:** 10.7554/eLife.14128

**Published:** 2016-01-22

**Authors:** Yi-Zhi Wang, Jing-Jing Wang, Yu Huang, Fan Liu, Wei-Zheng Zeng, Ying Li, Zhi-Gang Xiong, Michael X Zhu, Tian-Le Xu

Wang Y-Z, Wang J-J, Huang Y, Liu F, Zeng W-Z, Li Y, Xiong Z-G, Zhu MX, Xu T-L. 2015. Tissue acidosis induces neuronal necroptosis via ASIC1a channel independent of its ionic conduction. *eLife* **4**:e05682. doi: 10.7554/eLife.05682.

Published November 2, 2015

In the published article, a typographical error was introduced in the range of CP-1 in Figure 4A, which should have read 463-483 rather than 468-483.

The corrected Figure 4 is shown here:

**Figure:**
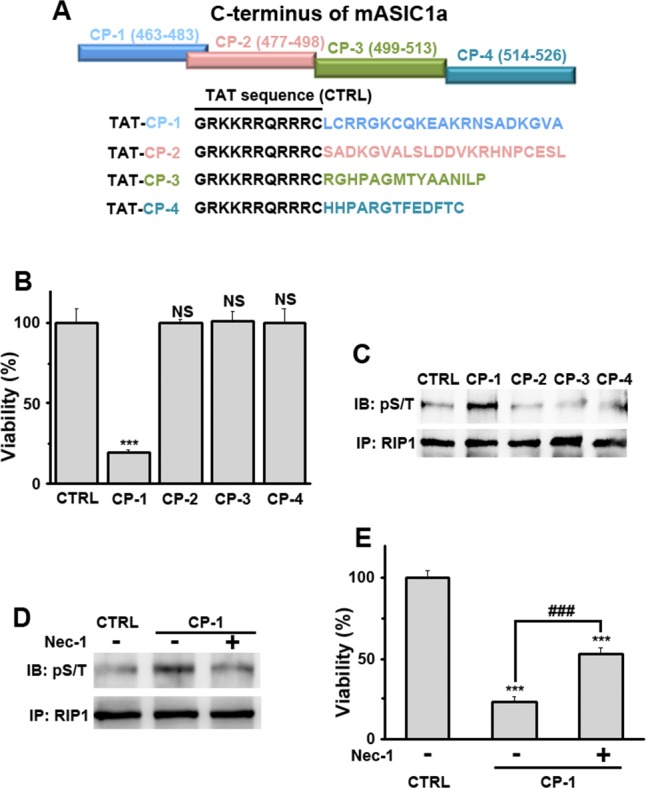

The originally published Figure 4 is also shown for reference:

**Figure:**
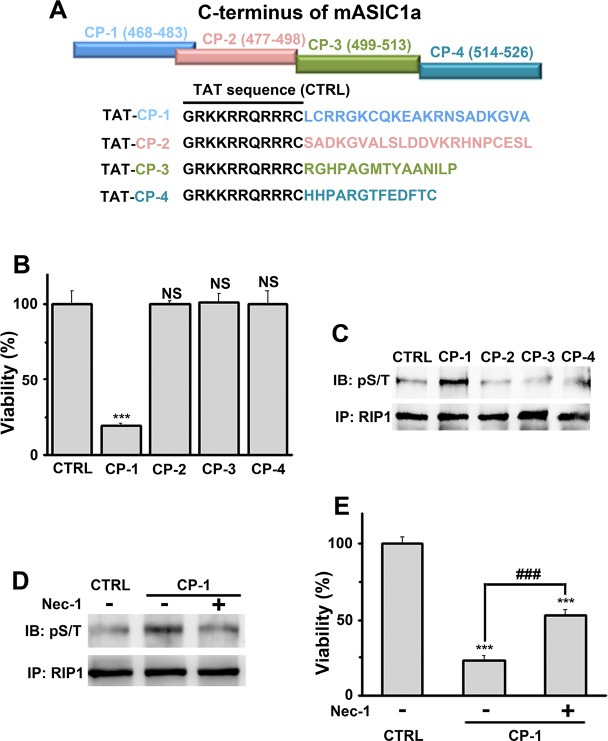

The article has been corrected accordingly.

